# High fidelity wastewater surveillance as an early warning tool for diphtheria in the German Ruhr metropolitan area

**DOI:** 10.3389/fpubh.2026.1891655

**Published:** 2026-09-08

**Authors:** Katharina Block, Josefa Welling, Alexander Thomas, Evelyn Heintschel von Heinnegg, Jan Kehrmann, Leah Consten, Adrian Dörr, Jule Gosch, Simon Magin, Jens Schoth, Jan Buer, Ivana Kraiselburd, Folker Meyer

**Affiliations:** 1 Institute for AI in Medicine, University Hospital Essen, University of Duisburg-Essen, Essen, Germany; 2 Department of Computer Science, University of Duisburg-Essen, Essen, Germany; 3 Department of Microbiology, University Hospital Essen, University of Duisburg-Essen, Essen, Germany; 4 Emschergenossenschaft (EGLV), Essen, Germany

**Keywords:** *Corynebacterium*, diphtheria, metagenomics, one health, public health, wastewater-based surveillance

## Abstract

**Introduction:**

Diphtheria is re-emerging as a public health concern in Europe, with recent fatalities underlining the need for strengthened surveillance. In highly vaccinated populations, limited clinical suspicion and reduced routine testing can obscure low-level circulation of toxigenic strains, increasing the risk of silent spread. In addition to classical *Corynebacterium diphtheriae* infections, the potential mobilization of the diphtheria toxin (*tox*) gene within and possibly beyond different *Corynebacterium* species represents a growing threat.

**Methods:**

We developed a high-fidelity wastewater-based surveillance approach capable of detecting *tox* genes as an early warning system to complement clinical and laboratory-based monitoring.

**Results and Discussion:**

During routine metagenomic surveillance, including weekly monitoring of the local wastewater treatment plant, screening with a *tox*-specific Hidden Markov Model (HMM) detected *tox* signals in 18 of 160 environmental samples. Technical and sample-availability constraints allowed PCR confirmation of 15 HMM-positive samples, and 14 of these PCR products were confirmed by amplicon sequencing. One detection coincided spatially and temporally with a *tox*-positive *Corynebacterium* clinical case: wastewater from the hospital ward and downstream treatment plant was positive that week and negative the following week. No corresponding clinical isolates were identified for the other wastewater signals. Although not conclusive, this concordance supports alerting clinicians and public-health authorities when *tox* is detected in wastewater.

## Introduction

Diphtheria, caused by toxigenic strains of *Corynebacterium diphtheriae* and related species, is a re-emerging, vaccine-preventable disease of growing concern in Europe (1, 2). In countries with long-standing vaccination programs, diphtheria is often perceived as a disease of the past and is therefore not routinely tested for, which may lead to underdiagnosis and delayed recognition of ongoing transmission. Recent outbreaks of *C. diphtheriae* in Berlin and Frankfurt, Germany, highlight the continued circulation of this pathogen and its capacity to cause clusters even in highly immunized populations, including cases with fatal outcomes (3). Beyond *C. diphtheriae*, several other species, including *Corynebacterium ulcerans* (4), *Corynebacterium pseudotuberculosis* (5), *Corynebacterium rouxii* (5) and *Corynebacterium ramonii* (6), have been recognized as potential carriers of diphtheria toxin (*tox*) gene, expanding the spectrum of species capable of producing diphtheria-like disease. Moreover, *Corynebacterium* strains classified as ‘nontoxigenic *tox* gene-bearing’ (NTTB) are also considered a potential risk (5). The expanding diversity of tox-harboring corynebacteria, together with accumulating evidence of increasing point mutations within the *tox* gene (7, 8), poses growing challenges for accurate diagnosis, molecular surveillance, and risk assessment.

From a public health perspective, diphtheria is of particular concern as a re-emerging but vaccine-preventable disease. Early detection of cases and signals of community circulation remains essential for timely outbreak response (9).

Wastewater-based epidemiology (WBE) has recently emerged as a sensitive and cost-effective surveillance tool, capable of capturing population-level signals of pathogen circulation independent of healthcare-seeking behavior, and of identifying pathogens that may otherwise go undetected in routine diagnostics (10). For diphtheria, WBE could provide an early warning system by detecting toxigenic corynebacteria in the community prior to widespread recognition of clinical cases. Due to historically high vaccination coverage in Germany, Diphtheria has become rare and its classic clinical presentation—a thick, grayish pseudomembrane on the tonsils, pharynx, nasal mucosa or grayish non-healing ulcers—is seldom encountered. Instead, patients may initially present with nonspecific symptoms, such as mild fever, sore throat, and general malaise, which complicates early recognition (11) and underscores the value of complementary surveillance approaches, such as WBE.

Metagenomics is a comprehensive method that captures the microbial diversity and resistance genes in complex samples, enabling the simultaneous detection of a wide range of known and unexpected targets without prior assumptions about their presence. This is particularly advantageous for pathogens such as toxigenic *Corynebacterium* spp., which may circulate at low prevalence and escape routine clinical testing. However, metagenomic detection in wastewater is inherently influenced by the rank-abundance structure of microbial communities, in which many clinically relevant pathogens occur at very low relative abundance (12). Consequently, low-abundance organisms or genes may be underrepresented in sequencing data, fragmented across contigs, or fall below detection thresholds when using conventional assembly-based approaches. Additionally, as the diphtheria *tox* gene is integrated in the bacterial chromosome via recombination mediated by corynebacteriophages (13), its position within the chromosome is variable. This genomic mobility further complicates assembly-based metagenomic analyses.

Targeted methods such as PCR offer higher sensitivity for specific pathogens but require *a priori* target selection and may miss genetically diverse or unexpected variants. The integration of sensitive profile-based screening methods, such as Hidden Markov Models (HMMs), with metagenomic data can partially mitigate these limitations by enabling the detection of conserved functional signatures even in fragmented or low-coverage sequences.

In this study, we developed and applied a targeted HMM-based pipeline to detect tox-positive *Corynebacterium* species in metagenomic datasets from wastewater. We combined this open discovery approach with PCR validation to assess the feasibility and reliability of wastewater-based surveillance for toxigenic Corynebacteria. Using metagenomic data we try to identify additional hosts for the corynebacterial *tox* gene by following the WHO recommendation of using the RNA polymerase β-subunit (*rpoB*) gene (14, 15) for host species determination. By integrating metagenomic screening and targeted molecular confirmation, our work contributes to the development of early warning systems for diphtheria and supports the broader use of wastewater monitoring as a public health surveillance tool.

## Methods

### Wastewater sampling

A total of 160 wastewater samples were collected during several research projects, from a variety of locations within the Ruhr metropolitan area, between November 2022 and August 2024 (see Supplementary Figure 1). This was not a comprehensive spatiotemporal survey, samples arose from routine and project-based surveillance, including weekly WWTP monitoring. Collection points included the inflow of the Klärwerk Emscher Mündung (KLEM) wastewater treatment plant, processing sewage from approximately 1.39 million residents, municipal sewer system sites representing populations ranging from 33,932 to 292,012 individuals (F, J, R, S), as well as sewers located at different clinics of the University Hospital Essen (KK, OZII, RLK). Additional samples correspond to unconnected wastewater treatment plants from the northern Ruhr metropolitan area (KLBT, KLDI, KLDU). An explanation of the sample site abbreviations can be found in Supplementary Table 1. More details on the sampling sites proximal to the University Hospital Essen are available in Thomas et al. (16) and Schmiege et al. (17).

Where possible, mixed 24-h composite samples were taken, using automated mobile samplers (Liquistation CSF48, Endress+Hauser). Alternatively, grab samples were used. Because grab samples and 24-h composite samples represent different temporal sampling strategies, results obtained using these approaches are not directly quantitatively comparable. Following collection, samples were transported to the laboratory, where they were stored at 4 °C overnight to allow for sedimentation.

### DNA extraction and metagenomic sequencing

DNA was extracted from 200 mL of sedimented wastewater by filtering through 0.45 μm pore size electronegative filters (MF-Millipore). The subsequent extraction of nucleic acids was carried out using the innuPREP AniPath DNA/RNA Kit, in accordance with the manufacturer’s protocol, operated on an InnuPure C16 touch device (Analytik Jena). DNA concentrations were then quantified using the Qubit 4 dsDNA HS Assay. Sequencing libraries were prepared using the Nextera XT DNA Library Prep kit, following the manufacturer’s instructions. The molarity of the libraries was assessed using both Qubit 4 dsDNA HS measurements and the Agilent Technology 2,100 Bioanalyzer (D1000 HS). Libraries were diluted to 2 nM and pooled. Shotgun metagenomic sequencing was conducted on the Illumina NextSeq 2000 platform using P3 and P4 (300 cycle) cartridges.

### Bioinformatic workflow

An average of 150 million reads per sample were generated. Adapter trimming and quality filtering were performed using fastp (18) and read quality was assessed using FastQC (19). Reads shorter than 30 base pairs or with a PHRED quality score below 20 were discarded. Subsequently, human reads were removed by mapping against a reference genome (GRCh38) with minimap2 (20). High-quality reads were assembled into contigs using MEGAHIT (21), with a minimal contig length of 300 bp. Protein-coding sequences were predicted using Prodigal with ‘meta’ option (22). For functional screening, Hidden Markov Models (HMMs) corresponding to diphtheria toxin-associated protein families were obtained from the PFAM database (PF02764, PF01324, and PF02763). These HMMs were used to scan the metagenomic datasets, allowing the identification of candidate sequences with similarity to the *tox* gene. Predicted proteins were screened separately against PF02764, PF01324, and PF02763 using hmmsearch with an e-value threshold of 0.001 (HMMER version 3.4).

### PCR validation

PCR assays were employed to amplify multiple conserved regions of the diphtheria *tox* gene. In parallel, amplification of the housekeeping gene *rpoB* was performed according to WHO recommendations to enable species-level discrimination among *Corynebacterium* spp. harboring the *tox* gene, including *C. diphtheriae*, *C. ulcerans/C. pseudotuberculosis*, *C. ramonii*, *C. rouxii*, *C. belfantii*, and *C. silvaticum* (14, 15). It should be noted that *C. ulcerans* and *C. pseudotuberculosis* cannot be differentiated based on *rpoB* sequence alone. All primers used in this study are listed in Table 1. Each PCR run included negative controls (extraction blanks and nuclease-free water) and positive controls consisting of a toxigenic *C. ulcerans* provided by the Institute of Medical Microbiology, University Medicine Essen.

**Table 1:** Primers used for *tox* gene detection and rpoB-based species identification.

Primer	Target gene	Sequence (5′–3′)	Reference position	Amplicon length (bp)	Source
*tox* gene detection					
PP1_fw	*C. diphtheriae tox (R-domain)*	CGTAATGGGCATTGCAGACG	K01722.1: 1,246–1,266	210	This work
PP1_rv		GGAGAATACGCGGGACGATT	K01722.1: 1,436–1,456		
PP2_fw	*C. diphtheriae tox (T&R-domain)*	GACTGACGAAGGTTCTCGCA	K01722.1: 570–590	935	This work
PP2_rv		CCAACTGACAGCATACCCGT	K01722.1: 1,485–1,505		
PP3_fw	*C. diphtheriae tox (C-domain)*	GAAAACTTTTCTTCGTACCACGGGACTAA	K01722.1: 353–382	248	(23)
PP3_rv		ATCCACTTTTAGTGCGAGAACCTTCGTCA	K01722.1: 573–602		
PP4_fw	*C. diphtheriae tox (R-domain)*	ATACTTCCTGGTATCGGTAGC	K01722.1: 991–1,011	297	(23)
PP4_rv		CGAATCTTCAACAGTGTTCCA	K01722.1: 1,267–1,287		
PP5_fw	*C. diphtheriae tox (T-domain)*	GGCCAAGATGCGATGTATG	NC_002935: 189,573–189,591	100	(24)
PP5_rv		CCCAATCAAGATTTATGCATGAC	NC_002935: 189,650–189,672		
rpoB-based species identification					
PP6_fw	*C. diphtheriae rpoB*	CGCCAGCAAGAAGAGCT	NC_002935: 409,927–409,943	120	(24)
PP6_rv		AGGCTCAGAAAGAGACAGC	NC_002935: 410,028–410,046		
PP7_fw	*C. ulcerans/C. pseudotuberculosis rpoB*	TAGATTCCTTCGCATGGCTCA	NC_017317: 405,526–405,546	135	(24)
PP7_rv		CGGAATAATCCTGAATCGGAG	NC_017317: 405,640–405,660		

Reference coordinates and expected amplicon sizes are shown.

### Sequencing of PCR products

Amplicons were sequenced to confirm *tox* gene identity. Following purification, PCR products were prepared for sequencing using the Oxford Nanopore Technologies (ONT) ligation sequencing kit with native barcoding. End-repair, adapter ligation, and library clean-up were performed in accordance with the manufacturer’s instructions. The prepared libraries were then loaded onto R10.4.1 flow cells and sequenced on a Nanopore GridION platform.

After sequencing, PCR products were analyzed using BLASTx (25) against the latest NCBI non-redundant (nr) database. This sequencing enabled the characterization and comparison of the *tox* genes present, facilitating the detection of genetic variations such as point mutations.

### Identification of all potential hosts for the *tox* gene

To identify possible host organisms carrying the *tox* gene contained in all wastewater samples, the *rpo*B gene was used as a marker, recognizing that it does not always allow distinction between all *Corynebacterium* species (15). For this, contigs containing *rpo*B genes were identified from metagenomics data by comparison against a custom DIAMOND database comprising all available full-length *rpo*B protein sequences from UniProt (128 sequences, database built in June 2025), using DIAMOND version 2.1.12 in ‘ultra-sensitive’ mode (26). *C. ramonii*, *C. belfantii* and *C. silvaticum* were not available for download and therefore are not included in the set of 128 corynebacterial *rpo*B genes. The database and additional information regarding its construction, including the commands used for database generation and analysis, are publicly available at GitHub: https://github.com/IKIM-Essen/WasteWater_Pathogen_Surveillance. For each sample, the highest bitscore per species was retained and visualized as a heatmap without the application of an abundance normalization. Clustering of potential host species was performed using the clustermap function of seaborn, with default settings on the obtained bitscores. This analysis was intended as a broad inventory of *Corynebacterium* lineages rather than physical linkage of *rpoB* and *tox* on the same contig.

## Results

### Detection of diphtheria *tox* gene in wastewater metagenomes

To assess the occurrence and distribution of Diphtheria toxin-associated sequences in wastewater, metagenomic data from samples across the northern Ruhr metropolitan area were systematically screened using PFAM Hidden Markov Models targeting conserved *tox* protein domains and positive hits were evaluated based on HMM bitscores. This analysis yielded 18 positives out of a total of 160 wastewater samples, with maximum bitscores for the detected HMM hits ranging from 25 to 81 (Table 2). Here, an HMM-positive sample denotes a sample containing at least one retained hit to any of the three *tox* gene domain HMM profiles, where retained hits met the e-value thresholds of 0.001. All positive samples contained hits exclusively to the PF01324 profile, corresponding to the R domain of the *tox* gene and no hits were detected for the other two domain profiles.

**Table 2:** Results of *tox* gene detection, *rpoB* PCR, and *tox*-amplicon sequencing in wastewater samples selected by HMM-based screening.

Sample ID	KK2427	F2402	F2406	F2409	F2410	J2401	J2406	S2401	S2402	S2405	S2406	S2410	KLEM2431	KLBT20230309	RLK2402	RLK2404	KLDU20230309	KLDI2433
Number	1	2	3	4	5	6	7	8	9	10	11	12	13	14	15	16	17	18
PCR_*tox*	+	−	+	+	+	+	+	+	+	+	+	−	+	+	+	+	+	n/A
PCR_*rpo*B	PT/U	−	PT/U	PT/U	−	−	PT/U	PT/U	PT/U	PT/U	−	−	−	PT/U	−	PT/U	−	N/A
PCR_seq_tox	+	N/A	−	+	+	+	+	+	+	+	+	N/A	+	+	+	+	+	N/A
HMM bitscore	81	30	26	31	27	31	25	27	36	28	35	35	26	27	31	30	26	31

PCR_tox, PCR assay targeting the *tox* gene; PCR_rpoB, species identification by rpoB PCR; PT/U, *C. pseudotuberculosis* and/or *C. ulcerans* detected; PCR_seq_tox, sequencing of the tox PCR amplicon; HMM bitscore, maximum score of the best HMM hit over all three models with e-value ≤ 0.001; KK, RLK, University Hospital Essen sewer; F, J, S, intra-urban sewer; KLEM, wastewater treatment plant connected to University Hospital Essen; KLBT, KLDU, KLDI, unconnected wastewater treatment plants.

Details of sampling site abbreviations are listed in Supplementary Table 1.

These *in silico* findings were further validated *in vitro* using PCR targeting the *tox* gene. Multiple primer sets targeting different regions of the gene, including sequences corresponding to the C, T and R domains, were evaluated. Consistent amplification of sample-derived DNA was obtained only with primers targeting the C domain of *tox* (PP3 primer pair in Table 1). This suggests that the C domain is less prone to genetic variation compared to other regions.

Of the 18 HMM-positive samples, 15 were confirmed through triplicate PCR, while two showed weak or inconsistent bands upon repeated amplification (Table 2). This study was performed retrospectively, on stored DNA obtained in the context of different investigations. One sample had already been exhausted at the time of PCR analysis. The weak/inconsistent amplification in the two remaining cases is likely due to sample degradation. To assess the sensitivity of the approach, a random subset of 17 from the remaining 142 HMM-negative samples was selected as negative controls for PCR analysis. PCR testing for the *tox* gene in these controls yielded no bands of the target product length.

Sequencing of the PCR products (Table 2) confirmed the validity of the approach, with 14 samples yielding high-quality reads that matched the expected genomic regions and the *tox* gene in BLASTX searches against the NCBI NR database. One PCR-positive sample did not generate usable sequencing reads, likely due to insufficient amplicon quantity or loss during library preparation and was therefore excluded from downstream sequence-based analyses. Supplementary Figure 2 shows the alignment of the amplicon sequences to a reference gene of *C. diphtheriae tox*.

The HMM screen and PCR interrogated different representations of the target: profile HMMs were applied to translated predicted proteins, whereas PCR amplified a defined nucleotide region. Consequently, the two approaches are not directly comparable on a one-to-one sequence basis. The present analysis did not compare HMM-positive protein sequences with PCR amplicon sequences, assess whether sequence divergence influenced PCR amplification, or evaluate whether the presence or number of HMM hits correlated with PCR success. In our dataset, all retained HMM hits corresponded to the PF01324 profile, whereas no retained hits were identified for PF02763 or PF02764. Therefore, we cannot draw conclusions regarding the relationship between HMM detection and PCR amplification success or the causes of the two discordant PCR results.

Because neither this metagenomic screening nor *tox*-targeted PCR alone allows reliable assignment of *tox* genes to a specific host species, *rpoB* PCR was performed as a species-discriminatory marker in accordance with WHO guidelines (14, 15). Within the analyzed sample subset, none of the detected *tox* genes were associated with *C. diphtheriae*. Instead, *rpoB* PCR results indicated the presence of *C. pseudotuberculosis* or *C. ulcerans* in nine samples (Table 2). In an additional eight samples, *rpoB* PCR excluded *C. diphtheriae*, *C. pseudotuberculosis*, and *C. ulcerans*.

The metagenomic *rpoB* screen and *rpoB* PCR were not designed as equivalent assays. The metagenomic analysis retained the highest species-level bitscore across all *rpoB*-containing contigs, whereas PCR tested only the specified diagnostic targets. Consequently, the two methods are not directly comparable on a sample-by-sample basis. In addition, neither approach physically links an identified *rpoB* sequence to a *tox*-containing contig. Therefore, a formal concordance analysis between metagenomic species assignments and rpoB PCR results was not performed.

### Possible *tox* gene hosts identified in wastewater samples

To further explore the broader taxonomic context of potential tox-harboring organisms, all metagenomic contigs containing the *rpo*B gene were analyzed across wastewater samples, irrespective of the presence of the *tox* gene. As *rpo*B serves as a conserved phylogenetic marker for *Corynebacterium* spp., this approach captures the full diversity of corynebacterial lineages that could, in principle, acquire a tox-encoding prophage. Clustering of the *rpo*B-containing contigs revealed three distinct groups with differing abundance patterns.

**Figure 1:**
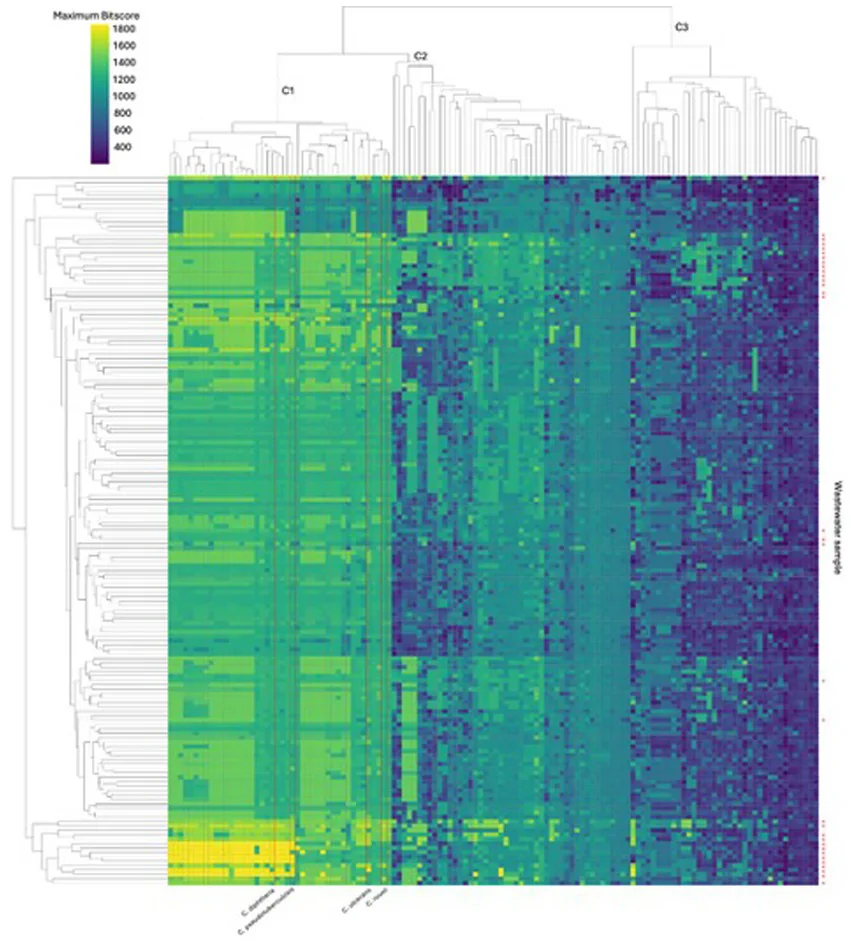
Clustered heatmap of *rpo*B gene sequences from 160 wastewater metagenomes from the northern Ruhr metropolitan area. Three distinct clusters of species are depicted on the x axis. Cluster 1 contains host-associated pathogens (mostly human and avian), including known carriers of the *tox* gene which are marked in red. Cluster 2 includes environmental/soil/dairy species and some human commensals. Cluster 3 contains aquatic or unusual host species as well as some human opportunistic pathogens. Supplementary Table 2 shows a detailed description of the *Corynebacterium* species contained in each cluster. Samples marked with a red star correspond to hospital wastewater.

Cluster 1 corresponds to human associated corynebacteria and constitutes the most abundant cluster identified in our samples (Figure 1). This includes all known carriers of the *tox* gene (*C. diphtheriae*, *C. ulcerans*, *C. pseudotuberculosis* and *C. rouxii*). The other two clusters correspond to environmental, dairy, soil, and human skin/commensals (cluster 2) and aquatic, marine, animal commensals and human opportunistic pathogens (cluster 3).

Although no clear separation by sample type was observed, subtle patterns were visually evident, with hospital wastewater samples showing greater similarity to each other, forming two defined clusters on the *Y*-axis of Figure 1. Hospital samples (marked with a red star in Figure 1) in general contain a higher abundance of species from cluster 1 than samples from intra-urban sewersheds and wastewater treatment plants, which integrate urban discharges together with additional environmental inputs.

As a substantial fraction of tox-positive samples did not yield *rpoB* PCR results consistent with *C. diphtheriae*, *C. ulcerans*, or *C. pseudotuberculosis*, the identity of the corresponding host species remains unresolved. Metagenomic *rpo*B profiling indicates that several species within cluster 1 (Figure 1), which comprises human-associated *Corynebacterium* spp. including known *tox* carriers, may represent potential hosts of the *tox* gene. Together, these findings point to a broader spectrum of possible tox-associated *Corynebacterium* species than those routinely targeted by clinical diagnostics.

### Patient-derived samples matching wastewater-based findings

In 2024, a tox-positive diphtheria case was identified at University Hospital Essen. Wastewater samples analyzed in this study included effluent from the hospital unit in which the patient was treated. This sample tested positive for the *tox* gene and excluded the *C. diphtheriae* as carrier (sample 1 in Table 2) and was collected 3 days after laboratory confirmation of the tox-positive *C. ulcerans* infection in the patient, while samples from before the hospitalization were negative.

A corresponding *tox* gene signal was also detected in wastewater from the inlet of the local wastewater treatment plant (sample 13 in Table 2) on the same date. The catchment area of this treatment plant serves approximately one million inhabitants. No *tox* gene signal was detected at other sampling time points from this treatment plant. Wastewater from both the hospital ward and the treatment plant was negative in the following week.

Together, these observations indicate a temporal association between clinical identification of a *tox*-positive case and the detection of *tox* gene signals in hospital and downstream municipal wastewater, illustrating the potential of wastewater-based surveillance to provide near real-time insights into diphtheria circulation. One of the 18 detections was plausibly attributable to the clinical case. No corresponding clinical isolates were identified for the remaining signals. Concordant positivity at the ward and downstream plant, followed by negative results at both sites the next week, strengthens the association without establishing causality or predictive performance. Future *tox* detections should therefore be treated as early surveillance signals warranting targeted clinical and public-health assessment.

## Discussion

In this work, we developed and applied a method capable of identifying diphtheria *tox* genes in metagenomic wastewater samples. Our approach was validated by both in-vitro PCR assays and sequencing of PCR amplicons, as well as comparison with clinical case records, underlining its potential robustness for integration into ongoing surveillance systems. Technical and sample-availability limitations allowed PCR confirmation of 15 of 18 HMM-positive samples, 14 amplicons yielded sequence confirmation. None of the 17 HMM-negative controls were PCR-positive. One wastewater detection was plausibly explained by the contemporaneous clinical case, whereas no matching clinical isolates were identified for the other signals. These results support a surveillance function but do not establish formal sensitivity, specificity, or predictive performance.

The method provides the opportunity to track potential mobilization of the *tox* gene across *Corynebacterium* species or even beyond the genus. This is of relevance in the context of horizontal gene transfer and the possibility of toxigenicity emerging in atypical hosts within the genus *Corynebacterium*. The clinical significance of such potential toxigenicity in atypical hosts remains unknown. Moreover, detection of the *tox* gene alone does not establish the bacterial host or demonstrate toxin expression. The incorporation of a sequence-based workflow further ensures adaptability: should genetic modification reduce the effectiveness of current primers, metagenomic data can be used to inform primer redesign, thereby maintaining assay sensitivity over time. In fact, our findings suggest limitations in the reliability of some currently used primers, as reproducible amplification was achieved only with primers targeting the C region of the *tox* gene.

Our present workflow is assembly-based, with profile HMMs used as the annotation step following open-reading-frame prediction. Accordingly, the relevant comparison is between HMM-based protein annotation and alternative annotation strategies rather than between assembly and HMMs. Although we did not perform a direct benchmark against nucleotide read mapping or pairwise sequence similarity searches (e.g., BLAST or DIAMOND), profile HMMs are well suited to detecting divergent or fragmented homologs because they model position-specific conservation across protein families. Similar approaches have previously been applied to wastewater metagenomes for the discovery of divergent plasmid, viral, and protein families (27–29). To our knowledge, no prior longitudinal wastewater-surveillance study has specifically targeted the diphtheria *tox* gene, making this an early proof-of-concept rather than a mature routine assay.

*C. diphtheriae* is primarily sought in respiratory and cutaneous specimens, and fecal detection is not part of standard diagnostic algorithms (14, 15). Wastewater *tox* signals therefore cannot be assumed to reflect fecal shedding and may derive from respiratory secretions, wound washing, hospital effluent, or other human or animal sources.

Several challenges remain: detecting low-abundance targets in complex wastewater metagenomes requires careful optimization of thresholds, as well as continued cross-validation with clinical and epidemiological data. These limitations highlight the need for ongoing refinement of both bioinformatic pipelines and laboratory assays to ensure reliable signal detection. Formal analytical performance and broader clinical concordance require longitudinal validation. The use of both grab and 24-h composite samples also limits direct quantitative comparison across samples.

Given the re-emergence of diphtheria in parts of Europe and its status as a vaccine-preventable disease, early detection of circulation signals is of clear public health relevance. Our findings support the inclusion of diphtheria toxin (*tox*) genes and their potential host species within wastewater-based epidemiology frameworks. By integrating untargeted metagenomic screening with targeted molecular validation, wastewater surveillance can provide timely, complementary information for public-health authorities and strengthen early-warning and preparedness strategies for diphtheria. Additionally, it could substantially improve preparedness: the ability to detect circulation of toxigenic *Corynebacterium* at the population level, independent of healthcare-seeking behavior, would complement clinical and laboratory-based surveillance, thereby strengthening the ability to respond rapidly to re-emerging infectious disease threats. The HMM screen can be added to routine analysis; confirmed *tox* signals should prompt targeted clinical and public-health assessment. Operationally, the HMM screen adds limited incremental work when deep shotgun metagenomes are already generated, but the complete workflow is not a low-complexity stand-alone assay because it depends on sequencing, assembly, protein prediction, profile searching, confirmatory PCR, and amplicon sequencing.

The success of PCR validation indicates that PCR can also be used to ascertain the presence of the tox gene, albeit not its sequence or taxonomic origin, therefore routine PCR for diphtheria toxin genes is an option to the public sector to get ahead of the curve.

## Data Availability

The datasets presented in this study can be found in online repositories. The names of the repository/repositories and accession number(s) can be found at: https://github.com/IKIM-Essen/WasteWater_Pathogen_Surveillance.
