# Supplementary material for: High fidelity wastewater surveillance as an early warning tool for diphtheria in the German Ruhr metropolitan area
Source: Front Public Health. 2026 Sep 8;14:1891655. doi: 10.3389/fpubh.2026.1891655 (PMC13593791; doi:10.3389/fpubh.2026.1891655)
Supplement: Supplementary file 1 [file Data_sheet_1.docx]

## **Supplementary Information**

## **Supplementary Table 1**: Sampling site abbreviations used throughout the manuscript. Sites are grouped into wastewater treatment plants, clinical wastewater locations, and intra-urban subsewersheds.

| **Category** | **Abbreviation** | **Sampling site** | **Description** |
| --- | --- | --- | --- |
| Wastewater treatment plant  (WWTP) | KLBT | Kläranlage Bottrop | WWTP Bottrop |
|  | KLDI | Kläranlage Dinslaken | WWTP Dinslaken |
|  | KLDU | Kläranlage Duisburg | WWTP Duisburg |
|  | KLEM | Klärwerk Emscher-Mündung | WWTP Emscher-Estuary |
| Clinical wastewater | KK | Kinderklinik | Pediatric clinic |
|  | RLK | Ruhrlandklinik | Specialized pulmonary hospital |
|  | OZII | Operatives Zentrum II | Surgical center |
| Intra-urban subsewersheds | F | Frohnhauserweg |  |
|  | J | Jahnstrasse |  |
|  | R | Ripshorsterstrasse |  |
|  | S | Sulterkamp |  |

## **Supplementary Table 2**: *Corynebacterium* species identified in the clustered *rpo*B gene sequences derived from 160 wastewater metagenomes from the northern Ruhr metropolitan area. Species are grouped according to the three clusters shown in a heatmap in Figure 1: Cluster 1 comprises predominantly host-associated pathogens (mainly human and avian); Cluster 2 contains mostly environmental species, and Cluster 3 contains aquatic or uncommon host-associated species.

| **Clade 1** | | **Clade 2** | | **Clade 3** | |
| --- | --- | --- | --- | --- | --- |
| *C. urogenitale* | *C. pseudotuberculosis* | *C. liangguodongii* | *C. singulare* | *C. mendelii* | *C. genitalium* |
| *C. anserum* | *C. vitaeruminis* | *C. riegelii* | *C. argentoratense* | *C. propinquum* | *C. humireducens* |
| *C. falsenii* | *Candidatus C. gallistercoris* | *C. suranareeae* | *C. frankenforstense* | *C. otitidis* | *C. doosanense* |
| *C. pelargi* | *C. urealyticum* | *C. glutamicum* | *C. wankanglinii* | *C. phocae* | *C. hadale* |
| *C. pseudopelargi* | *Candidatus C. intestinavium* | *C. flavum* | *C. renale* | *C. courieae* | *C. marinum* |
| *C. pygosceleis* | *C. auriscanis* | *C. efficiens* | *C. variabile* | *C. yonathiae* | *C. testudinoris* |
| *C. antarcticum* | *C. resistens* | *C. gallinarum* | *C. capitovis* | *C. pseudogenitalium* | *C. comes* |
| *C. meridianum* | *Candidatus C. faecigallinarum* | *C. crudilactis* | *C. lowii* | *C. tuberculostearicum* | *C. afermentans* |
| *C. gerontici* | *C. kroppenstedtii* | *C. endometrii* | *C. oculi* | *C. lizhenjunii* | *C. sundsvallense* |
| *C. macclintockiae* | *C. pseudokroppenstedtii* | *C. lujinxingii* | *C. maris* | *C. phoceense* | *C. aquatimens* |
| *C. evansiae* | *C. treponotabidum* | *C. pseudodiphtheriticum* | *C. callunae* | *C. incognita* | *C. imitans* |
| *C. jeikeium* | *C. yudongzhengii* | *C. pollutisoli* | *C. coyleae* | *C. tapiri* | *C. hylobatis* |
| *C. bovis* | *C. freneyi* | *C. cystitidis* | *C. pyruviciproducens* | *C. pilbarense* | *C. uterequi* |
| *C. durum* | *C. poyangense* | *C. glaucum* | *C. felinum* | *C. meitnerae* |  |
| *C. amycolatum* | *C. uropygiale* | *C. halotolerans* | *C. mucifaciens* | *C. tuscaniense* |  |
| *C. sphenisci* | *C. ulcerans* | *C. alimapuense* | *C. confusum* | *C. lipophilum* |  |
| *C. xerosis* | *C. glyciniphilum* | *C. choanae* | *C. accolens* | *C. timonense* |  |
| *C. hindrelae* | *C. kalidii* | *C. occultum* | *C. striatum* | *C. mycetoides* |  |
| *C. aquilae* | *C. rouxii* | *C. pilosum* | *C. sp.* | *C. thomssenii* |  |
| *C. mustelae* | *C. spheniscorum* | *C. haemomassiliense* | *C. ammoniagenes* | *C. auris* |  |
| *C. diphtheriae* |  | *C. ureicelerivorans* | *C. glucuronolyticum* | *C. lipophiloflavum* |  |
| *C. epidermidicanis* |  | *C. kutscheri* | *C. camporealensis* | *C. qintianiae* |  |
| *C. matruchotii* |  | *C. minutissimum* | *C. casei* | *C. macginleyi* |  |
| *C. canis* |  | *C. aurimucosum* |  | *C. flavescens* |  |

**Supplementary Figure 1**: Overview of wastewater sampling sites and sampling timeline. (A) Map showing the locations of the investigated wastewater treatment plants (WWTPs) and clinical wastewater sampling sites in North Rhine-Westphalia, Germany. Clinical wastewater sites OZII and KK discharge into the KLEM WWTP catchment, following the given intra-urban trajectory (F, J, S, R). (B) Monthly number of wastewater samples collected at each sampling site included in this study.

**Supplementary Figure 2**: Coverage of the amplified and sequenced *tox* gene regions aligned to the Corynebacteriophage beta diphtheria toxin gene (GenBank: K01722.1, length: 1942 bp), confirming correct target amplification. The amplified region is located in the diphtheria *toxin C-domain* (position 353-602 bp).
